# Molecular evidence of neuroinvasive Sindbis virus infection in humans: detection in cerebrospinal fluid by next generation sequencing

**DOI:** 10.1080/22221751.2026.2703397

**Published:** 2026-07-11

**Authors:** Maria Grazia Cusi, Gianni Gori Savellini, Chiara Cassol, Cesira Nencioni, Laura Bernini, Danilo Tacconi, Giulia Alessandri, Letizia Rizzo, Gabriele Anichini, Teemu Smura, Olli Vapalahti

**Affiliations:** a Department of Medical Biotechnologies, University of Siena, Siena, Italy; b Microbiology and Virology Unit, Santa Maria delle Scotte University Hospital, Siena, Italy; c Infectious Diseases Unit, Ospedale Della Misericordia, Grosseto, Italy; d Infectious Diseases Unit, Ospedale San Donato, Arezzo, Italy; e Department of Virology, Faculty of Medicine, University of Helsinki, Helsinki, Finland; f Department of Veterinary Biosciences, Faculty of Veterinary Medicine, University of Helsinki, Helsinki, Finland; g Virology and Immunology, Helsinki University Hospital Diagnostic Center, Helsinki, Finland

**Keywords:** Alphaviruses, sindbis virus, Differential diagnosis, CNS infection, mNGS

## Abstract

Sindbis virus (SINV) is a mosquito-borne alphavirus causing seasonal outbreaks in northern Europe, Africa, and Russia. Neurological involvement in humans is poorly documented, and detection in cerebrospinal fluid (CSF) has not previously been reported. This study provides the first unequivocal evidence of human CNS involvement by detecting SINV RNA directly in the cerebrospinal fluid of four autochthonous patients presenting with acute neurological symptoms in south eastern Tuscany, Italy, July–August 2025. Utilizing metagenomic Next Generation Sequencing (mNGS), we reconstructed complete viral genomes, strongly supporting a causal relationship between SINV and neurological manifestations. Phylogenetic analysis revealed a complex epidemiological landscape in Italy characterized by the co-circulation of Clade D lineages. Our findings fundamentally expand the clinical spectrum of SINV, demonstrating that it is not merely an arthritogenic pathogen, but a neuroinvasive threat to humans. This highlights the critical need to include SINV in the differential diagnosis of viral CNS infections in endemic areas and underscores the urgency for enhanced European laboratory surveillance.

## Introduction

Sindbis virus (SINV) is an enveloped, single-stranded, positive-sense RNA virus belonging to the family *Togaviridae* and is primarily transmitted by *Culex* spp. mosquitoes [1,2]. SINV is maintained in enzootic transmission cycles involving ornithophilic mosquitoes and migratory birds, which play a key role in the long distance dissemination of viral lineages across Africa, Europe, and Asia [3,4]. In humans, infection is classically associated with febrile illness accompanied by rash and persistent arthralgia, and SINV is an established cause of seasonal outbreaks in northern Europe, Africa, and Russia [3, 5-7]. In endemic northern European regions, SINV infections are historically linked to arthritogenic syndromes such as Pogosta disease in Finland and Ockelbo disease in Sweden, where periodic epidemic waves have been associated with ecological and climatic conditions favouring vector amplification [8]. In contrast, the epidemiology of SINV in Southern Europe remains poorly understood. Serological, entomological, and veterinary studies suggest that the virus circulates more widely throughout Mediterranean countries than currently appreciated, including Italy, Spain, Greece, and the Balkans, although human infections are only rarely diagnosed [9-12]. Italy represents a particularly relevant ecological setting because of its position along major migratory bird flyways connecting sub-Saharan Africa with continental Europe. Previous studies have documented evidence of SINV circulation in mosquitoes, birds, and horses in different Italian regions, supporting the existence of local enzootic transmission cycles [9]. However, the burden of human disease and the genetic diversity of circulating strains remains largely unexplored.

We report four autochthonous cases of SINV infection presenting with neurological manifestations that occurred in south-eastern Tuscany, Italy, in July and August 2025. To date, detection of SINV in cerebrospinal fluid (CSF) has not been documented in humans, and neurological involvement has primarily been demonstrated in animal models [12]. In this study, we applied an unbiased metagenomic next-generation sequencing (mNGS) approach to identify and characterize the full viral genome directly from CSF samples, thereby providing molecular evidence of SINV associated central nervous system infection in humans. The aim of this study is to raise awareness of the potential occurrence of SINV outbreaks in countries where the virus is known to circulate, but it is not routinely investigated as an aetiological agent of human infection during the warm season. Our findings underscore the importance of broader and more systematic surveillance strategies, including molecular testing in patients presenting with unexplained neurological syndromes in endemic areas.

## Materials and methods

### Case descriptions and sampling

During July-August 2025, CSF samples from patients admitted to Misericordia Hospital (Grosseto, Italy; GR) and San Donato Hospital (Arezzo, Italy; AR) with acute onset neurological symptoms, including lower limb asthenia, subjective vertigo, nausea and photophobia, suggestive of meningitis or encephalitis, were referred to the Microbiology and Virology Unit, Policlinico Santa Maria alle Scotte (Siena), for investigation of neurotropic viral infections. CSF chemical–physical analysis was consistent with aseptic meningitis. First and second line microbiological investigations were performed.

### Virological real-time RT–PCR

Viral RNA was purified from clinical specimens by using the EZ1 DSP virus kit (Qiagen, Milan, Italy). Real-time Reverse-Transcription quantitative Polymerase Chain Reaction (RT-qPCR) was performed for neurotropic pathogens, including herpes simplex virus types 1 and 2, varicella zoster virus, Epstein–Barr virus, cytomegalovirus, chikungunya virus, dengue virus, tick-borne encephalitis virus, influenza A and B viruses, enteroviruses, West Nile virus, and Toscana virus (TOSV). Three CSF specimens that tested negative for known neurotropic viruses and one that resulted positive for TOSV (Case 3) were subsequently analyzed by SINV RT-qPCR using the TaqPath RT-qPCR mix (Applied Biosystems, Milan, Italy), including primers and probe specific for the SINV Nsp1 gene [13]. Reactions were carried out on Applied Biosystems™ 7500 Real-Time PCR System (Applied Biosystems). All tested samples resulted positive for SINV.

### Metagenomic next generation sequencing

Metagenomic Next Generation Sequencing (mNGS) was performed on CSFs, and the library preparation was carried out with the VSP v2 (Illumina S.r.l., Milan, Italy) according to the manufacturer’s instructions. Libraries were loaded on a V2 micro MiSeq 300-cycle flow cell (Illumina S.r.l.). FastQ Files were analyzed with the BaseSpace ™ platform by Illumina with the Dragen targeted microbial app (Illumina S.r.l.ly). The analysis identified SINV mNGS in all samples, with a mean genome coverage of 99.95%, a mean depth of 710X, and a mean of 76.850 aligned reads and homology to reference sequences ranging from 99.93% to 99.95%, confirming the unequivocal presence of the virus.

### Phylogenetic analysis

Phylogenetic tree was constructed using the maximum-likelihood method implemented in IQ-TREE 2 [14]. All complete Sindbis virus coding sequences were downloaded from GenBank and aligned using MAFFT v7.526 [15]. Potentially recombinant sequences and sequences showing less than 0.1% divergence were removed prior to analysis. The best-fit substitution model GTR + F + R3 was selected using ModelFinder [16] and applied for tree inference. Genotype and clade annotations follow those proposed elsewhere [17–19]. The viral genome detected in each sample (11.527 base length) was subsequently analyzed using BLAST against the GenBank database to assess homology and to assign each sequence to currently circulating reference strains.

### Serological investigation for Sindbis antibodies

Recombinant Sindbis virus E2 antigen (Immune Technology, New York, NY, USA) was used to set up an in-house ELISA test for the detection of virus-specific IgG and IgM. HRP-conjugated anti-human IgG or IgM (Jackson ImmunoResearch, Cambridgeshire, United Kingdom) was used as secondary antibodies and TMB as substrate. Absorbance at 450 nm was read using the VICTOR Nivo spectrophotometer (Revvity). Samples were considered positive for OD_450_ > 0.2. A serum from a SINV infected person from Finland was used as a positive control.

## Results

### Case 1

**Figure 1.**
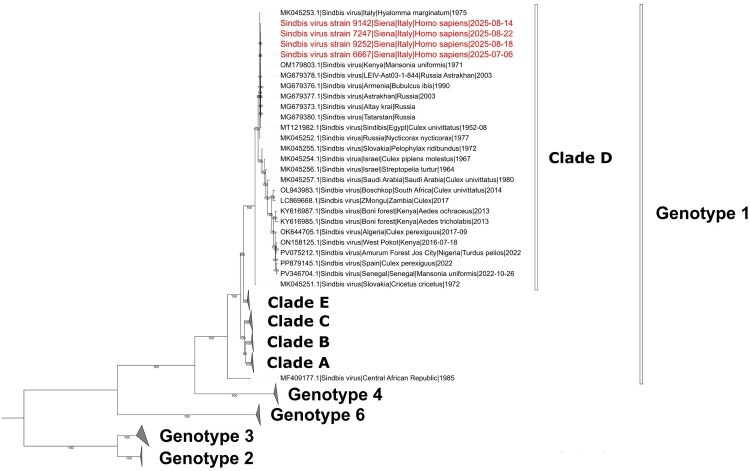
Phylogenetic analysis of the SINV strains identified in south eastern Tuscany, Italy, July-August 2025. A phylogenetic tree was constructed using the maximum-likelihood method, including all complete Sindbis virus coding sequences available from GenBank and sequences from Italian clinical samples (in red). The sequenced samples have a length ranging from 11,482 bp to 11,527 bp with a mean genome coverage of 99.95%. All samples clustered within clade D.

A 51-year-old woman was admitted in August 2025 for evaluation of persistent fever, severe headache, and transient mental confusion following a recent stay at the seaside resort of Senigallia, Italy. Approximately 12 days before admission, shortly after returning from Senigallia, she developed a progressively worsening headache associated with fever and the onset of confusion refractory to paracetamol, nonsteroidal anti-inflammatory drugs (NSAIDs), and corticosteroids prescribed by her general practitioner. She also reported burning pain localized to the right frontal region and, on the day of admission, burning pain involving the soles of both feet. No skin rash or exanthematous lesions were observed. Arterial blood gas analysis and routine blood chemistry were within normal limits. Brain computed tomography was negative, while electroencephalography showed nonspecific abnormalities. Lumbar puncture revealed clear CSF with elevated protein concentration (745 mg/L), leukocyte count of 18 cells/µL with mononuclear predominance (94.4%), polymorphonuclear cells 5.6%, and erythrocytes 1000 cells/µL. CSF bacterial cultures and molecular testing were both negative. On the third hospital day, serum serology resulted in a positive test for IgG to West Nile virus, prompting patient isolation and further investigations. Reverse-Transcription Polymerase Chain Reaction (RT–PCR) testing for West Nile virus and other arboviruses performed on blood and urine samples were negative. Molecular testing on CSF for herpes simplex virus types 1 and 2, West Nile virus, and Toscana virus was also negative. A specific RT–PCR for SINV performed on a CSF sample allowed the detection of the viral genome. An mNGS confirmed the presence of the SINV. BLAST analysis of the SINV genome sequence showed the highest similarity to a previously reported Russian strain. (Acc. No. MG679378.1) (Figure 1 and Table 1). During hospitalization, the patient received intravenous fluid and electrolyte replacement, NSAIDs, and corticosteroid therapy, with progressive clinical improvement and resolution of symptoms. She was discharged home in stable condition after a week.

**Table 1.** Summary of clinical virological data of SINV cases.

Case (Accession No.)	Hospitalization	Days from symptoms to sampling	IgM OD	IgG OD	Real-Time PCR on CSF	TOSV/SINV RT-qPCR Ct	SINV reference (% of homology)
1 (PZ055499)	San Donato Hospital; Arezzo (AR)	4	0.208	0.610	Positive SINV	29	Russia Acc. No. MG679378.1 (99.93%)
2 (PZ055497)	Misericordia Hospital; Grosseto (GR)	4	N.D.	N.D.	Positive SINV	32	Kenya Acc. No. OK644705.1 (99.95%)
3 (PZ246733)	Misericordia Hospital; Grosseto (GR)	7	0.635	0.546	Positive TOSV + SINV	27/30	Kenya Acc. No. KY616985.1 (99.95%)
4 (PZ055498)	Misericordia Hospital; Grosseto (GR)	3	ND	ND	Positive SINV	31	Kenya Acc. No. KY616985.1 (99.95%)

Clinical and virological data of SINV cases (n = 4) collected in Italy during July-August 2025 from patients admitted to Misericordia Hospital (Grosseto, Italy; GR) and San Donato Hospital (Arezzo, Italy; AR) and results from diagnostic and mNGS investigations. GenBank submitted sequence lengths: PZ055499 (11,527 bp); PZ055497 (11,527 bp); PZ246733 (11,527 bp); PZ055498 (11,527 bp). Virus specific antibodies were considered negative when OD_450_ < 0.2 and positive when OD_450_ > 0.2.

N.D.: not done

Serological investigation on serum drawn three weeks after the appearance of symptoms demonstrated the presence of specific SINV IgM and IgG, by ELISA, using the recombinant E2 protein as antigen (Table 1).

### Case 2

A 58-year-old man with a medical history of arterial hypertension, allergic asthma, and chronic sinus disease presented to the Emergency Department of Grosseto Hospital in August 2025 with a frontal and supraorbital headache of acute onset, associated with fever and asthenia, lasting for one week, partially responsive to non-steroidal anti-inflammatory drugs. In the Emergency Department, a non-contrast brain computed tomography (CT) scan was performed and was negative for intraparenchymal lesions or acute cerebrovascular events. Laboratory tests demonstrated a mild elevation of C-reactive protein (1.08 mg/dL). Neurological examination did not show overt meningeal signs, except for a minimal bulbocavernosus reflex. In light of the clinical presentation, a lumbar puncture was performed and empirical antibacterial and antiviral therapy was initiated. CSF chemical–physical analysis revealed clear fluid, glucose concentration of 57 mg/dL, elevated protein level of 70 mg/dL, lactate concentration of 2.4 mmol/L, and marked pleocytosis of 253 cells/mm³ with a predominance of mononuclear cells (90%). Multiplex RT–PCR testing on CSF for herpes simplex virus types 1 and 2, varicella-zoster virus, Epstein–Barr virus, chikungunya virus, tick-borne encephalitis virus, enteroviruses, and West Nile virus and Toscana virus was negative, as were bacterial and fungal cultures. Given the negativity of first-line microbiological investigations, a specific RT-qPCR for SINV performed on a CSF sample allowed the detection of the viral genome, subsequently confirmed by mNGS. The SINV genome was compared with the data bank

and the sequence was shown to be most closely related to an African strain (Acc. No. OK644705.1) (Figure 1 and Table 1). During hospitalization, the patient showed progressive clinical improvement, with marked reduction of headache, defervescence, and normalization of inflammatory markers. After 8 days of hospitalization, he was subsequently discharged home.

### Case 3

A 36-year-old woman with a medical history notable for fibromyalgia and recurrent headache disorders was admitted to Grosseto Hospital in July 2025 because of acute-onset, progressively worsening headache and fever for six days. The presenting symptoms were associated with lower limb asthenia, subjective vertigo, nausea, and photophobia. Upon evaluation in the Emergency Department, a non-contrast computed tomography (CT) scan of the brain was performed and showed no evidence of acute intracranial pathology. Neurological examination revealed mild nuchal rigidity accompanied by subtle signs of meningeal irritation. In view of the clinical suspicion of central nervous system infection, hospital admission was disposed and a lumbar puncture was performed. CSF analysis was consistent with aseptic meningitis. The chemical–physical analysis of the CSF demonstrated a glucose concentration of 54 mg/dL, elevated protein levels of 72 mg/dL, a lactate concentration of 2.3 mmol/L, and pleocytosis of 46 cells/mm³ with a marked predominance of mononuclear cells (95%). Initial multiplex RT–PCR testing on CSF yielded negative results. CSF cultures, as well as RT–PCR assays for other neurotropic pathogens, including herpes simplex virus types 1 and 2, varicella-zoster virus, Epstein–Barr virus, chikungunya virus, tick-borne encephalitis virus, influenza A and B viruses, enteroviruses, West Nile virus and polyomaviruses, were all negative. A specific RT–PCR for Toscana virus and SINV performed on the CSF sample allowed the detection of both viral genomes. An mNGS confirmed the co-infections of these emerging pathogens. The SINV genome was compared with the data bank and the sequence was shown to be most closely related to a Kenyan strain (Acc. No. KY616985.1) (Figure 1 and Table 1). The Magnetic Resonance Imaging (MRI) of the brain did not reveal any pathological findings. The patient showed a progressive and favorable clinical course, characterized by defervescence and a gradual decline in inflammatory markers until complete normalization. After a total hospital stay of seven days, the patient was discharged home in stable clinical condition. Serological investigation on serum drawn three weeks after the appearance of symptoms demonstrated the presence of specific SINV IgM and IgG, by ELISA, using the recombinant E2 protein as the antigen (Table 1).

### Case 4

An 84-year-old woman affected with arterial hypertension, bilateral glaucoma, and atrial fibrillation on non-vitamin K antagonist oral anticoagulant therapy was admitted to the Emergency Department of Grosseto Hospital on August 21st, 2025, because of a fluctuating confusional state and progressively worsening psychomotor slowing, associated with olfactory hallucinations. No fever was reported throughout the clinical course. The patient had been neurologically intact prior to symptom onset. Initial laboratory investigations revealed a marked elevation of C-reactive protein (15 mg/dL) associated with monocytosis. In the emergency setting, a non-contrast brain computed tomography (CT) scan was performed and did not show evidence of acute intracranial lesions. Electroencephalography (EEG) demonstrated diffuse, intermittent triphasic waves on a mildly slowed background rhythm, findings considered compatible with encephalitis. Neurological consultation recommended brain magnetic resonance imaging (MRI) with gadolinium contrast and infectious diseases evaluation. Given the ongoing anticoagulant therapy, lumbar puncture was deferred for 48 hours, and empirical broad-spectrum antibacterial and antiviral therapy was initiated. Subsequent CSF examination documented unremarkable results, with clear appearance and normal glucose concentration, protein levels, lactate, and cell count. First and second-line microbiological investigations were negative. In particular, urinary antigen tests for *Legionella pneumophila* and *Streptococcus pneumoniae*, serological assays for *Mycoplasma pneumoniae* and *Chlamydia pneumoniae*, multiplex RT–PCR panels for upper respiratory tract pathogens and arboviruses, and two sets of blood cultures were all negative. CSF investigations, including routine cultures, FilmArray testing, cryptococcal antigen, and PCR assays for herpes simplex virus types 1 and 2, varicella-zoster virus, Toscana virus, tick-borne encephalitis virus, West Nile virus, Chikungunya virus, and enteroviruses, were negative. Microscopy, PCR, and culture for *Mycobacterium tuberculosis* on CSF were also negative. Despite the absence of microbiological confirmation, antiviral therapy with acyclovir was continued for a total of 14 days in consideration of the neurological and electroencephalographic findings. During hospitalization, brain MRI with contrast enhancement did not reveal acute pathological changes, while serial EEG recordings demonstrated progressive improvement. At discharge, the patient was persistently afebrile, alert, oriented, and neurologically returned to baseline status comparable to her pre-admission condition. Retrospective investigation by a specific RT–PCR for SINV performed on a CSF sample allowed the detection of the viral genome, further confirmed by mNGS. The SINV genome was compared with the data bank and the sequence was shown to be most closely related to a Kenya strain (Acc. No. KY616985.1) (Figure 1 and Table 1).

### Phylogenetic analysis of Italian SINV strains

Phylogenetic analysis at the nucleotide level was performed to establish the viral clade of the Italian SINV strains, considering the different geographical origins of the patients who tested positive. Phylogenetic analysis indicated that all four samples belonged to genotype 1, clade D, having only 1 nucleotide (nt) difference (*p*-distance = 0.008%) between each other (Figure 1) but no amino acid differences (*p*-distance = 0%) (Table 2). Notably, the SINV strain identified in the patients at Misericordia Hospital in Grosseto (GR), located on the Tyrrhenian coast, differed from that detected in the patient at San Donato Hospital in Arezzo (AR), who had recently vacationed on the Adriatic coast of Italy (Table 1). The sequence differences between the Siena strains and the closest BLAST matches OM179803.1|Sindbis_virus|Kenya|Mansonia_uniformis|1971 and MG679378.1|Sindbis_virus|LEIV-Ast03-1-844|Russia_Astrakhan|2003 were further investigated. Within the open reading frame (ORF), four amino acid differences (Table 2) (*p*-distance = 0.106%) and 5–6 nucleotide differences (*p*-distances = 0.04–0.05%) were observed between the Siena strains and OM179803.1|Sindbis_virus|Kenya|Mansonia_uniformis|1971. Similarly, seven amino acid differences (*p*-distance = 0.186%) (Table 2) and 8 nucleotide differences were found between the Siena strains and MG679378.1|Sindbis_virus|LEIV-Ast03-1-844|Russia_Astrakhan|2003 (*p*-distance = 0.07%) (Figure 1 and Table 1).

**Table 2.** Amino-acidic differences between Siena SINV strains and relative reference sequences.

Viral gene	Amino acid position	Siena (Italy)	OM179803 (Kenya; Africa)	MG679378 (Astrakhan; Russia)
Nsp1	127	T	T	A
	411	I	T	T
	536	A	A	T
Nsp2	1255	T	N	T
E2	2920	N	N	D
	2972	S	S	G
	3039	E	E	K
	3074	E	K	E
6k	3326	V	V	A
E1	3462	T	I	T

Major amino-acidic differences among Italian SINV strains and relative reference sequences were highlighted. Among the Siena strains, there are no amino acid differences, while a total of 10 amino-acid (a.a.) substitutions were identified targeting the Nsp1/2, E1/2, and 6k viral proteins, distributed as 4 and 7 a.a. differences between Siena strains and OM179803 (Sindbis virus|Kenya|Mansonia uniformis|1971) and MG679378 (Sindbis virus|LEIV-Ast03-1-844|Russia Astrakhan|2003), respectively.

## Discussion

The process by which Sindbis virus (SINV) reaches the Central Nervous System (CNS) remains incompletely resolved, despite decades of investigation, particularly in animal models. Following mosquito inoculation, viral replication initially occurs in peripheral tissues, including dendritic cells, fibroblasts, and muscle-associated cells, leading to a transient viremia that enables systemic dissemination [20]. Several mechanisms of CNS entry have been proposed, including the direct infection of cerebral endothelial cells, which results in localized disruption of tight junction integrity and increased blood–brain barrier (BBB) permeability [7]. Alternatively, cytokine induced BBB dysfunction may compromise endothelial stability, thereby facilitating viral penetration into neural tissue, while another compelling hypothesis involves the “Trojan horse” migration of infected leukocytes across the BBB [21]. Although evidence for this latter mechanism is less definitive in SINV than in flaviviral infections [22], leukocyte trafficking clearly contributes to CNS inflammatory responses and may facilitate viral dissemination under specific physiological conditions. Once inside the CNS, experimental evidence indicates that SINV can replicate in human neuronal cells and induce caspase-dependent apoptosis, characterized by increased caspase-3 and Bax expression alongside decreased Bcl-2 levels [23]. Neurons constitute the principal target cell population during SINV encephalitis, and experimental models have consistently demonstrated a pronounced age dependent susceptibility, with immature neurons being substantially more permissive to infection than mature ones [23]. However, neuronal death is not exclusively driven by direct cytopathic effects; immune-mediated injury also plays a pivotal role, as activated microglia, infiltrating T lymphocytes, and proinflammatory cytokines can amplify neuronal dysfunction even as viral replication declines [24]. Furthermore, recent transcriptomic studies have revealed substantial heterogeneity among infected neuronal populations [25], where some neurons rapidly activate antiviral programmes and survive infection, while others exhibit transcriptional collapse and progress towards apoptosis, a heterogeneity now regarded as a major determinant of clinical outcome. Although extensive research is still required to fully characterize these neuropathogenic mechanisms directly in humans, animal models have historically served as the gold standard to demonstrate the marked neurotropism of SINV. In murine models, SINV targets CNS neurons and triggers immunopathological responses that heavily influence disease severity, such as type I interferon responses (IFN alpha/beta) which, while essential for viral control, modulate inflammatory pathways that dictate clinical outcomes [26]. Interestingly, while BBB infection is traditionally linked to neuroinvasion in alphaviruses [21], murine data show that SINV crosses the BBB prior to overt barrier disruption, suggesting that CNS entry precedes clinically detectable damage [22,27], while also mirroring age-dependent differences, showing fatal infections in neonates versus non-fatal encephalomyelitis in adults. A critical knowledge gap has persisted regarding the relevance of SINV neuroinvasion in humans. While experimental studies have consistently demonstrated marked neurotropism in animal models, evidence in human disease has remained scarce and largely indirect [28]. In the present study, SINV RNA was detected directly in the cerebrospinal fluid of four patients presenting with neurological manifestations, and viral genome identification was further supported by metagenomic next-generation sequencing. These findings provide strong evidence that SINV can reach the human central nervous system and are consistent with a potential role of the virus in neurological disease. Nevertheless, our findings should be interpreted with caution. Detection of viral RNA in CSF demonstrates neuroinvasion, but does not, by itself, establish that SINV was the sole cause of the neurological manifestations observed. Although extensive microbiological investigations were performed, the contribution of additional infectious or non-infectious factors cannot be completely excluded. This limitation is particularly relevant in Patient 3, in whom both SINV and Toscana virus RNA were detected in CSF. Given the well-established neurotropism of Toscana virus, the relative contribution of each virus to the clinical presentation cannot be determined. Therefore, this case should be interpreted as evidence of viral co-detection within the central nervous system rather than definitive proof of SINV attributable disease.

Taken together, our observations support the hypothesis that SINV may be associated with neurological disease in humans and provide evidence of its ability to invade the central nervous system. This finding was further strengthened by metagenomic Next Generation Sequencing (mNGS), which enabled the reconstruction of the complete viral genome, strongly supporting a causal association between SINV infection and neurological manifestations. Beyond confirming causality, our genomic data reveal crucial insights into the epidemiological significance of SINV circulation in Italy. All Italian strains clustered within a closely related group together with previously described Kenyan, Egyptian, Russian and Armenian strains, indicating limited genetic divergence within this lineage and a largely intermixed topology without clear geographic segregation. This pattern is consistent with the relatively slow evolutionary rate of SINV, estimated to be on the order of 10^−^⁵ substitutions per site per year (approximately 5 × 10^−^⁵ in previous whole-genome analyses [17]), which results in limited genetic divergence over time. Notably, a historical Italian strain from 1975 occupies a basal position within this sub-cluster, suggesting that similar viral lineages may have been present in Italy for several decades. This topology does not support a simple geographic partitioning of lineages but rather suggests long-term circulation and evolutionary continuity across a broad geographic range. The observed differences between strains from Grosseto and Arezzo, therefore, likely reflect small-scale genetic variation within a widely distributed lineage rather than the co-circulation of divergent strains. Notably, the identified strains belong to SINV genotype I (gt1) Clade D, which is phylogenetically distinct from the classical arthritogenic SINV gt1 clade A historically associated with Pogosta and Ockelbo disease in Northern Europe [29]. Therefore, although our findings demonstrate the presence of Clade D strains in human CSF, further investigations are required to determine whether the observed neurotropism represents a broader feature of SINV biology or a lineage specific aspect associated with Clade D. Interestingly, in a recent Spanish study [28] SINV gt1 clade D strain was found in the CSF of a Spanish pediatric patient through metagenomics, however, because only limited viral genomic material was recovered, the finding remained suggestive rather than definitive. Our findings significantly expand the known clinical spectrum of SINV, showing that it is not only an arthritogenic pathogen. Indeed, hallmark features of SINV gt1 clade A infection, such as arthralgia and rash, were absent in our patients, supporting its role as a true neuroinvasive threat. Taken together, our observations support the hypothesis that SINV may be associated with neurological disease in humans and provide evidence of its ability to invade the central nervous system. However, larger studies integrating clinical, virological, immunological, and epidemiological data will be required to define the frequency, spectrum, and causal contribution of SINV to neurological syndromes. Therefore, SINV should be considered in the differential diagnosis of viral CNS infections, and further studies are warranted to clarify its pathogenic role and true burden of neuroinvasive disease.

## Data Availability

Sequence data generated in this study have been submitted to GenBank under accession numbers PZ055497, PZ055498, PZ055499, and PZ246733.
